# Coping Styles for Mediating the Effect of Resilience on Depression Among Medical Students in Web-Based Classes During the COVID-19 Pandemic: Cross-sectional Questionnaire Study

**DOI:** 10.2196/25259

**Published:** 2021-06-07

**Authors:** Lina Zhao, Kristin Sznajder, Dan Cheng, Shimeng Wang, Can Cui, Xiaoshi Yang

**Affiliations:** 1 Institute of Foreign Languages China Medical University Shenyang China; 2 Department of Public Health Sciences College of Medicine Pennsylvania State University Philadelphia, PA United States; 3 Hospital of Stomatology China Medical University Shenyang China; 4 Department of Anesthesiology Shengjing Hospital of China Medical University Shenyang China; 5 Department of Social Medicine College of Health Management China Medical University Shenyang, Liaoning Province China

**Keywords:** resilience, coping styles, depression, medical students, COVID-19, coping, mediation, web-based education, e-learning, smartphone, cross-sectional

## Abstract

**Background:**

Due to strict, nationwide, comprehensive COVID-19 protective measures, including home quarantine, all Chinese medical students began taking web-based classes beginning in the spring semester of 2020. Home quarantine, web-based classes, and the stress surrounding the COVID-19 pandemic may have triggered an increased incidence of mental health problems among medical students. Although there have been increasing amounts of literature on depression among medical students, studies focusing on positive psychological resources, such as resilience during the COVID-19 pandemic, still need to be expanded.

**Objective:**

This study aims to assess depression among medical students who are taking web-based classes during the COVID-19 pandemic and to investigate the role of coping styles as mediators between resilience and depression.

**Methods:**

A cross-sectional study of 666 medical students involving stratified sampling in Shenyang, Liaoning Province, China, was completed between March 20 and April 10, 2020. The participants responded to a self-administered, smartphone-based questionnaire, which included the Patient Health Questionnaire-9, Simplified Coping Style Questionnaire, and Ego Resilience 89 Scale. Hierarchical linear regression and structural equation modeling were used in this study.

**Results:**

The prevalence of depression among the participants was 9.6% (64/666) in this study. The regression analysis revealed that grade (the year in which the medical student was in training) (*P*=.013), how well students adapted to web-based classes (*P*<.001), their levels of resilience (*P*=.04), and their coping styles were independent predictors for depression (*P*<.001). Resilience and positive coping styles were negatively related to depression (resilience: *P*=.04; positive coping styles: *P*<.001), and negative coping styles were positively related to depression (*P*<.001). The structural equation modeling analysis showed that the effect of resilience on depression was partially mediated by coping styles (*P*=.007).

**Conclusions:**

In this study, it was found that the prevalence of depression was slightly low and coping styles mediated the association between resilience and depression among medical students during the COVID-19 pandemic. These findings have significant implications for future studies. Future studies and interventions should aim to improve resilience and promote positive coping styles.

## Introduction

The COVID-19 pandemic has deeply affected peoples’ lives all over the world since its emergence in 2019. Both isolation and economic pressure have had a profound impact on the psychosocial environment in each affected country. The pandemic has also increased the public’s susceptibility to detrimental psychological consequences [1]. According to a Chinese study, more than half the study population reported moderate or severe psychological impacts resulting from the COVID-19 pandemic [2]. Quarantine-associated mental health issues involve depression, anxiety, and irritability [3].

School lockdowns were implemented in many countries [4]. In order to better prevent and control the further spread of COVID-19, many universities in China began conducting web-based classes in February 2020. With the unprecedented number of web-based classes, home-quarantined medical students' psychological status is an important area of investigation. It has been reported that the rate of anxiety and depression among medical students is high [5], and the COVID-19 pandemic might bring about further risks to their mental health. Medical courses usually involve practice and experiments. However, web-based classes cannot provide such opportunities, which may add to medical students' worries about their academic achievements and result in high amounts of mental stress. Students have been reported to prefer studying in classrooms over having web-based classes due to the feeling of being together during classroom learning and the ability to share perspectives [6]. Therefore, the lack of peer contact and face-to-face communication with teachers in web-based classes may increase the risk of developing worry, anxiety, or even depression among medical students.

Depression—a mood disorder defined by sadness, inactivity, the loss of appetite or overeating, and difficulty in concentrating—can result in the reduced ability to perform daily activities among some people. Depression is one of the most often identified health issues among college undergraduates [7,8] and is especially common among medical undergraduates [9,10]. Previous studies have found that approximately 30% of medical undergraduates in Europe experience anxiety or depression [11,12]. Mental health disorders among home-quarantined university students have also been found in a previous study, which reported that the prevalence of depression was as high as 9% among university students about 1 month after COVID-19 outbreak in China [13]. A large cross-sectional study that included 44,447 Chinese college students reported an overall prevalence of depression symptoms of 12.2% during the COVID-19 pandemic [14]. The increased prevalence of depression was also observed in the winter 2020 academic semester in a US study [15].

The cognitive-behavioral model of health anxiety [16] suggests that some individuals have maladaptive assumptions about their health and, consequently, tend to overconsume health information, which could lead to high levels of anxiety [17]. Based on this model, individuals who have high levels of anxiety tend to be more anxious during a pandemic [18,19]. The transactional stress model theory [20] states that the responses of individuals who are faced with stress are affected by the coping process during a stressful experience [21] and that situation appraisal and coping can be influenced by positive personal resources.

There are many recent, positive psychology [22,23] studies that focus on depression, and resilience—a positive capability—is a topic of wide concern [24,25]. Resilience refers to one’s capability to adjust to challenges and adverse events [26,27] such as trauma, threats, or other major stresses, and resilience may prevent depression [28-30]. People who are less resilient are more susceptible to pathological reactions to adversities, while people who are more resilient are more likely to be protected against adversities [31].

According to the transactional stress model theory, the process of coping plays a significant role in individuals’ responses to stress [21]. Coping presents strategies of cognition and behavior that individuals can use to master, decrease, or stand up to the inward or outward demands of stressful situations [32]. Dynamic reactions to adversities aid individuals in preventing themselves from developing psychological impairments. Coping styles generally consist of two categories—positive coping and negative coping. Positive coping involves managing problems, adjusting quickly to stressors, and allaying pressure, whereas negative coping includes avoidance, social withdrawal, and the pitying of oneself, which all exacerbate anxiety. Studies have shown the significant correlation between negative coping styles and depression [33,34]. Positive coping styles help individuals to cope with adversities actively. This may involve seeking others’ advice and finding out solutions to problems, which is beneficial for mental well-being. Previous studies have indicated that coping styles play a mediating role in the relationship between perfectionism and depression among undergraduate students [35] and mediate the association between depression and eating disorders among Chinese female undergraduates [36]. Thus, it has been speculated that coping styles would mediate the relationship between resilience and depression.

This study assessed the prevalence of depression among medical students and determined whether coping styles play a mediating role in the relationship between resilience and depression among medical students during the COVID-19 pandemic. This study examined the following three hypotheses: (1) higher levels of resilience predict lower depression scores; (2) coping exerts a positive effect on relieving depression; and (3) coping styles mediate the relationship between resilience and depression.

## Methods

### Study Design and Participants

A cross-sectional study involving stratified sampling was carried out by conducting a self-administered questionnaire on Wenjuanxing—a smartphone- and web-based questionnaire platform—between March 20 and April 10, 2020. Wenjuanxing is a widely used, open, web-based questionnaire platform that was developed by Changsha Ranxing Information and Technology Limited Company. The free and self-design version was used in this study. The validity and reliability of the questionnaire that we designed and used in this study were examined.

Medical students who were home-quarantined in their first, second, or third year at China Medical University were eligible for this study. In total, 8 classes from each grade in which the medical students were in training were randomly selected. The medical students from 24 classes in their first, second, or third year at China Medical University were selected as the participants and finished the questionnaire. Of the total 720 medical students who were recruited in this study, 666 participants responded completely to the questionnaire, resulting in a valid response rate of 92.5% (666/720).

### Ethics Statement

All participants were fully informed of the study protocol and provided informed consent prior to taking the web-based questionnaire. Participation was voluntary and anonymous. The study protocol was approved by the Ethics Committee of China Medical University.

### Demographic Characteristics of Participants

Demographic information, including grade (the year in which the medical student was in training; ie, freshman, sophomore, and junior year), gender, age (<20 years and ≥20 years), fathers’ education (junior middle school and below and specialized secondary school and above), mothers’ education (junior middle school and below and specialized secondary school and above), monthly income (≤RMB 5000 [US $778.30] and >RMB 5000 [US $778.30]), major (clinical medicine and others), and whether students adapted to web-based classes (yes or no), was collected.

### Measurement of Depression

Depression was measured with the Patient Health Questionnaire-9 (PHQ-9), which is commonly used for the measurement of depression based on the Diagnostic and Statistical Manual of Mental Disorders, Fourth Edition criteria, is comparably sensitive and specific, and includes 9 items [37]. A score of ≥10 on the PHQ-9 was considered as the indicator for the existence of depression. The PHQ-9 has been widely used in previous studies among the Chinese population [38]. Many previous studies have confirmed that the PHQ-9 has good reliability, ranging from 0.749 to 0.92 [38-41], and the Cronbach α coefficient of the PHQ-9 was .927 in this study.

### Measurement of Coping Styles

Coping styles were assessed with the Simplified Coping Style Questionnaire, which included 20 items using a Likert scale of 0 (never) to 3 (frequently). In this study, the Cronbach α coefficient of the Simplified Coping Style Questionnaire was .862.

### Measurement of Resilience

Resilience was measured with the Ego Resilience 89 Scale, which has good internal reliability [42] as well as superior construct validity [43]. Participants completed the 14-item, 4-point scale by indicating the degree to which they approved of each statement, with scores ranging from 1 to 4 (1=“does not apply at all”; 2=“applies slightly, if at all”; 3=“applies somewhat”; 4=“applies quite strongly”). In this study, the Cronbach α coefficient of the Ego Resilience 89 Scale was .935.

### Statistical Analysis

SPSS 17.0 (IBM Corporation) and AMOS (Analysis of Moment Structures) 24.0 (IBM Corporation) were used for statistical analyses in the present study. The comparison of differences among classified groups was conducted with *t* tests (two-tailed) and one-way analysis of variance tests, and a two-tailed *P* value of less than .05 considered statistically significant.

In order to examine the incremental variance of any given set of independent variables and to assess the mediating role of coping styles in the association between resilience and depression, hierarchical linear regression analysis was used. Depression was used as the dependent variable. Resilience and coping styles were used as the independent variables. The variables were entered into models via the following step-by-step process: in step 1, the demographic characteristics of the medical students were entered; in step 2, resilience was entered; and in step 3, coping styles were entered. The following criteria for establishing the mediating effects, according to Baron and Kenny [44] in their approach to analyzing mediation, are supposed to be met: (1) the independent variable (resilience) is significantly related to both the dependent variable (depression) and the mediator (coping styles); (2) the mediator (coping styles) is significantly related to the dependent variable (depression); and (3) the adding of the mediator (coping styles) in the model significantly lessens or clears away the independent variable's (resilience's) impact on the dependent variable (depression).

To prove that coping styles played a mediating role in the relationship between resilience and depression, structural equation modeling was used. Bootstrapping strategies were used to examine the mediating role (a×b product) of coping styles in the relationship between resilience and depression. The bootstrap estimate was based on 5000 bootstrap samples, and the bias-corrected and accelerated (BCa) 95% CI for each a×b product was examined. The goodness of fit was determined by the following: a chi-square to df ratio of <5, a goodness-of-fit index (GFI) of >0.90, a comparative fit index (CFI) of >0.90, a root mean square error of approximation (RMSEA) of <0.08, and a Tucker-Lewis Index (TLI) of >0.90.

### Ethical Approval

The study protocol conformed to the ethical standards of and was approved by the Ethic Committee of China Medical University. All participants gave their consent after being informed of the purpose and procedure of the study via a web-based platform. The confidentiality and anonymity of all participants' collected information were ensured.

### Informed Consent

The informed consent of every participant was acquired before the launch of the procedures of this research.

## Results

### Demographic Characteristics and Depression Distribution Among the Participants

The demographic characteristics and their respective mean depression scores are shown in Table 1. Approximately 41.7% (278/666) of study participants were freshmen. The average age of the participants was 20 years. Approximately 39.3% (262/666) of the participants were males. The students reported their fathers’ education and mothers’ education as junior middle school or below, accounting for 55.4% (369/666) and 59.9% (399/666) of the responses, respectively. About 49.2% (328/666) of the participants reported their family’s monthly income as more than RMB 5000 (US $778.30). Most students were studying clinical medicine (460/666, 69.1%). With respect to gender, the depression scores of the male students were significantly higher than those of the female students (*P*=.045). Up to 88.9% (592/666) of the participants were adapting to web-based classes, while 11.1% (74/666) were not. The depression scores among the students who were not adapting to web-based classes were significantly higher than those among the students who were adapting to web-based classes (*P*<.001).

**Table 1:** Demographic characteristics and the distributions of depression among students (N=666).

Variables		Value, n (%)	Depression score, mean (SD)
**Grade**			
	Freshman	278 (41.7)	4.31 (4.83)
	Sophomore and junior	388 (58.3)	3.67 (4.20)
**Gender**			
	Male	262 (39.3)	4.37 (5.12)^a^
	Female	404 (60.7)	3.66 (3.98)
**Age** **(years)**			
	<20	315 (47.3)	3.84 (4.48)
	≥20	351 (52.7)	4.02 (4.48)
**Fathers’ education**			
	Junior middle school and below	369 (55.4)	4.02 (4.69)
	Specialized secondary school and above	297 (44.6)	3.84 (4.21)
**Mothers’ education**			
	Junior middle school and below	399 (59.9)	3.99 (4.47)
	Specialized secondary school and above	267 (40.1)	3.86 (4.50)
**Monthly income (RMB [US $])**			
	≤5000 (US $778.30)	338 (50.8)	3.84 (4.16)
	>5000 (US $778.30)	328 (49.2)	4.04 (4.79)
**Major**			
	Clinical medicine	460 (69.1)	3.91 (4.54)
	Others	206 (30.9)	4.00 (4.35)
**Adapting to web-based classes**			
	Yes	592 (88.9)	3.44 (4.07)
	No	74 (11.1)	7.88 (5.54)^b^

^a^Significant at the .05 level (two-tailed).

^b^Significant at the .01 level (two-tailed).

### Correlations Between Depression and Continuous Variables

The correlations between depression and the continuous variables are shown in Table 2. Depression among medical students was significantly and negatively associated with both resilience (*P*<.001) and positive coping styles (*P*<.001), while depression was significantly and positively associated with negative coping styles among medical students (*P*<.001).

**Table 2:** The correlations among depression and continuous variables.

Variables^a^		Depression	Resilience	Positive coping styles	Negative coping styles
**Depression**					
	*r*	1	−0.288^b^	−0.332^b^	0.356^b^
	*P* value	—^c^	<.001	<.001	<.001
**Resilience**					
	*r*	−0.288^b^	1	0.558^b^	−0.089^b^
	*P* value	<.001	—	<.001	<.001
**Positive coping styles**					
	*r*	−0.332^b^	0.558^b^	1	0.078^b^
	*P* value	<.001	<.001	—	<.001
**Negative coping styles**					
	*r*	0.356^b^	−0.089^b^	0.078^b^	1
	*P* value	<.001	<.001	<.001	—

^a^The mean scores for depression, resilience, positive coping styles, and negative coping styles are 3.94 (SD 4.48), 43.88 (SD 7.77), 38.80 (SD 6.76), and 17.79 (SD 4.83), respectively.

^b^Significant at the .01 level (two-tailed).

^c^Not applicable.

The linear regression models of depression among medical students are presented in Table 3. The final regression model (model 3) explained 31% of the total variance in depression scores. Resilience and coping styles explained 7% and 13.5% of the total variance in depression scores, respectively. Grade (*P*=.013), resilience (*P*=.04), positive coping styles (*P*<.001), and negative coping styles (*P*<.001) were significant predictors for depression. Grade, resilience, and positive coping styles were negatively associated with depression, while negative coping styles were positively associated with depression.

**Table 3:** The hierarchical linear regression analysis of depression.

Variables		Depression, standardized β		
		Model 1^a^	Model 2^b^	Model 3^c^
**Block 1: demographic characteristics**				
	Grade (freshman vs sophomore and junior)	−.088	−.112^d^	−.103^d^
	Gender (male vs female)	−.029	−.040	.000
	Age (<20 years vs ≥20 years）	.051	.073	.079
	Fathers’ education (junior middle school and below vs specialized secondary school and above)	−.019	.018	.022
	Mothers’ education (junior middle school and below vs specialized secondary school and above)	−.019	−.022	−.019
	Monthly income (≤RMB 5000 [US $778.30] vs >RMB 5000 [US $778.30])	.043	.049	.041
	Major (clinical medicine vs others)	−.019	−.037	−.053
	Adapting to web-based classes (yes vs no)	.303	.270^d^	.197^e^
**Block 2: resilience**		—^f^	−.270^e^	−.087^d^
**Block 3: coping styles**				
	Positive coping style	—	—	−.287^e^
	Negative coping style	—	—	.335^e^

^a^The R^2^ and ΔR^2^ values of model 1 are 0.105 and 0.105, respectively.

^b^The R^2^ and ΔR^2^ values of model 2 are 0.175 and 0.070, respectively.

^c^The R^2^ and ΔR^2^ values of model are 0.310 and 0.135, respectively.

^d^Significant at the .05 level (two-tailed).

^e^Significant at the .01 level (two-tailed).

^f^Not applicable.

### The Mediating Role of Coping Styles in the Relationship Between Resilience and Depression

**Figure 1:**
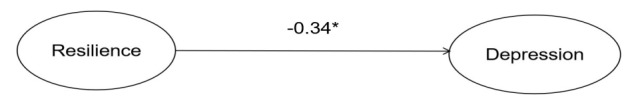
Standardized solutions for the structural equation model of resilience and depression. The standardized path coefficient is shown on the unidirectional arrow path. *The coefficient of the path is significant at the *P*<.05 level.

Figure 1 presents the direct effect of resilience on depression (c=−.34; *P*<.001) before coping styles were entered as a mediator. The model revealed that resilience had a significant negative effect on depression (*P*<.001), and this model had good model fit indices (chi-square to df ratio<5; RMSEA=0.052; CFI=0.962; GFI=0.928; adjusted GFI=0.904; TLI=0.954; Figure 1).

**Figure 2:**
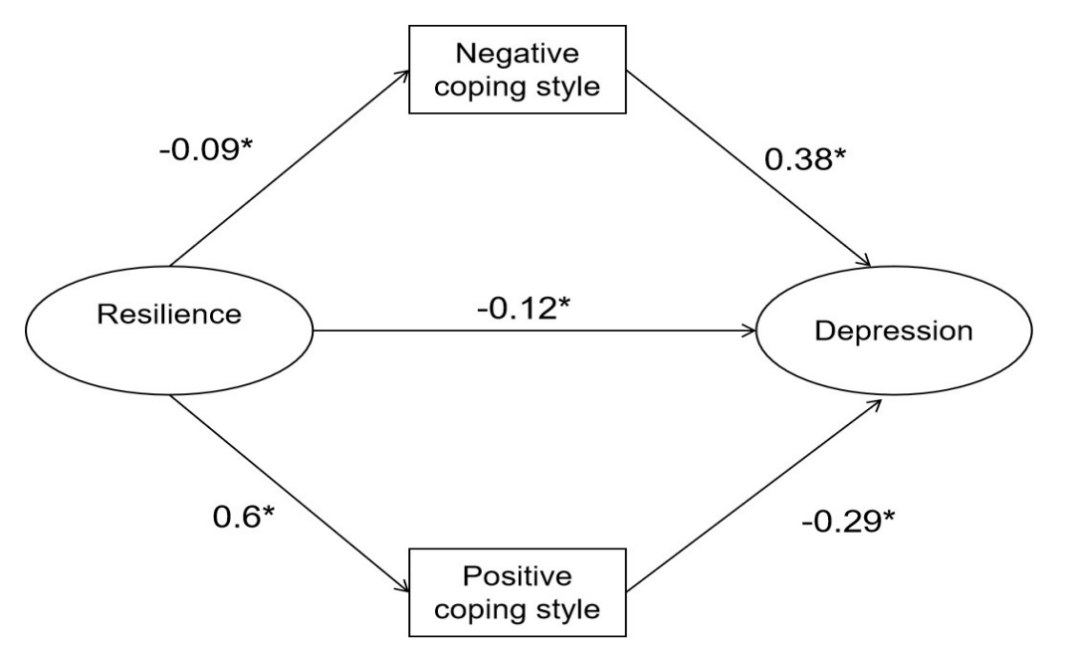
Structural equation modeling of the mediating role of coping styles in the relationship between resilience and depression. Standardized path coefficients are shown on the unidirectional arrow paths. *The coefficient of the path is significant at the *P*<.05 level.

Figure 2 represents the structural equation modeling of the mediating role of coping styles in the relationship between resilience and depression, and the standardized path coefficients are presented on the unidirectional arrow paths. When coping styles were used as the mediator, the path coefficient between resilience and depression decreased significantly (from −.34 in Figure 1 to −.12 in Figure 2; *P*=.007), which confirmed coping styles' partial mediating role in the association between resilience and depression. This model yielded a good model fit (chi-square to df ratio<5; RMSEA=0.051; CFI=0.957; GFI=0.923; adjusted GFI=0.900; TLI=0.949). According to the BCa bootstrap test, coping styles played a significant mediating role in the association between resilience and depression (*P*=.007; a×b=−0.22; BCa 95% CI −0.324 to −0.153), which proved that coping styles had a significant role as a mediator between resilience and depression.

## Discussion

As far as we know, this study presents the first attempt to investigate the relationship among resilience, coping styles, and depression among Chinese medical students in the context of web-based classes during the COVID-19 pandemic. In this study, 64 out of the 666 (9.6%) participants reported depression, which was slightly higher than the depression prevalence of 9% in a study of home-quarantined Chinese college students that was conducted during the COVID-19 pandemic [13] but lower than the prevalence rates in most previous studies. Such studies have indicated high prevalence rates of depression among college students (ranging from 12.2% to 25.3%) during the COVID-19 pandemic [14,45,46]. Depression is one of the most commonly occurring mental health issues among college students [47-49], and the prevalence of depression is especially high among medical students [10,50-52]. However, the prevalence of depression among medical students in this study was slightly lower than that of a Swedish study (12.9%) [53] and much lower than that of a study (25%) on US and Canadian medical students [10,54]. One possible reason why this study found lower rates of depression among Chinese medical students may be that staying with family helped ease symptoms of depression. This is good for mental health, and web-based classes provided the students with more opportunities to use available resources at home for entertainment, which might have helped with easing symptoms of depression. Furthermore, due to their medical knowledge, medical students may be more likely to perceive the pandemic objectively, which might prevent depression.

In this study, grade (*P*=.013) and whether students adapted to web-based classes (*P*<.001) had significant impacts on depression. With the national requirement of “suspending classes without suspension of learning” in China during the COVID-19 pandemic, web-based studying at home provided the only feasible method for keeping up with the learning schedule. The results in this study showed that the 11.1% (74/666) of participants who did not adapt to web-based classes had significantly higher depression scores (*P*<.001). This may be because the students who failed to adapt to web-based classes were more accustomed to face-to-face instruction and web-based classes added to their stress, which could result in increased levels of depression.

It was found that resilience was negatively related to depression in this study. With regard to our hypotheses, which were based on the transactional stress model theory [20], the findings from this study indicated that higher levels of resilience were predictive of lower levels of depression. This is in line with a substantial number of previous studies [55-58]. A prospective, multi-institutional study focusing on US medical students reported that resilient students were less susceptible to depression [59]. It was clarified in previous studies that resilience played a role in attenuating depression in different populations [56,57], such as college students and medical and nursing students [60,61]. Our study indicated that resilience played a protective role against depression, which is in line with prior studies [62,63]. Higher levels of resilience have been found to correlate with better subjective health [60], less distress [64], and more optimism among medical students [65]. Previous studies have also found that resilience among medical students might play a critical role in maintaining mental well-being during the COVID-19 pandemic. Web-based classes have been viewed as an intervention that plays a role in decreasing levels of anxiety associated with the pandemic [66]. However, there have been some students who might have had low resilience and failed to adapt to web-based classes well. Lower resilience has been found to be related to higher incidence rates for psychological issues [67,68]. Home-quarantined medical students who transitioned from learning in classrooms on campus to web-based learning at home might have experienced added stress, which might trigger depression. Resilience might help medical students adapt to uncertainty and maintain mental well-being while taking web-based classes at home during the COVID-19 pandemic. First, high resilience might help medical students combat the stressful situations of web-based classes, thus relieving the symptoms of depression. However, students with low resilience might be less likely to adapt to web-based classes well and, consequently, could be more susceptible to anxiety or even depression. Second, students with high levels of resilience might recover more quickly from adversities and cope with problems more actively, which could help with lowering their susceptibility to depression during the pandemic. Third, students who have high levels of resilience might be more likely to have successful experiences of coping with and recovering from adversities, including the pandemic.

The findings from this study showed that coping styles had a significant effect on relieving depression (*P*<.001), which is in line with previous studies [69,70]. Our study also showed that positive coping was inversely related to depression, while negative coping was positively related to depression among Chinese medical students. A positive coping style could help students cope with problems (eg, web-based classes) more rationally and might reduce stress, which in turn might prevent depression. This study also indicated that coping styles mediated the effect of resilience on depression. The coefficient of the resilience to depression path decreased after the coping style variables were added to the model, which indicated that coping styles had a partial mediating role in the relationship between resilience and depression. It is possible that individuals who are more resilient might be more likely to adopt a positive coping style, which might increase their likelihood of perceiving stressful situations, such as being quarantined at home while taking web-based classes during the COVID-19 pandemic, as surmountable. Therefore, they might be less likely to experience depression. Such individuals are also more likely to have better control over their emotions and be more motivated to figure out the solutions to problems, thus allowing them adapt to adverse situations. This could be beneficial for their mental well-being. Our results implied that positive coping and resilience training would be beneficial to medical students in terms of confronting the COVID-19 pandemic more positively and adapting to web-based classes more easily, which might help them to fight stress, reduce depression levels, and maintain mental well-being.

A few limitations exist in this study. First, this study is cross-sectional, which limits its ability to identify causal associations between variables. Second, self-reported measures may have resulted in response bias in this study. Third, the study sample only included medical students in years 1 to 3 from 1 university in northeastern China, which might limit the generalizability of our results.

### Conclusion

This study found that Chinese medical students in web-based classes experienced slightly low levels of depression during the COVID-19 pandemic. Resilience (*P*=.04) and coping styles (*P*<.001) were significantly related to depression. Positive coping styles played an essential role in decreasing depression levels among medical students, while negative coping styles were positively related to depression. It was also found that coping styles mediated the association between resilience and depression. This study indicates that interventions that aim to enable the development of positive coping styles among individuals and improve their resilience are of great practical importance to decreasing depression levels among medical students while they are taking web-based classes during the large-scale COVID-19 pandemic.

## Abbreviations

AMOS: Analysis of Moment Structures
BCa: bias-corrected and accelerated
CFI: comparative fit index
GFI: goodness-of-fit index
PHQ-9: Patient Health Questionnaire-9
RMSEA: root mean square error of approximation
TLI: Tucker-Lewis Index
